# A Novel Splice Site Variant in *COL6A1* Causes Ullrich Congenital Muscular Dystrophy in a Consanguineous Malian Family

**DOI:** 10.1002/mgg3.70032

**Published:** 2024-11-11

**Authors:** Alassane Baneye Maiga, Ibrahim Pamanta, Salia Bamba, Lassana Cissé, Salimata Diarra, Sidi Touré, Abdoulaye Yalcouyé, Seydou Diallo, Salimata Diallo, Fousseyni Kané, Seybou Hassane Diallo, Hamidou Oumar Ba, Cheick Oumar Guinto, Kenneth Fischbeck, Guida Landoure, Idrissa Ahmadou Cissé, The H3Africa Consortium

**Affiliations:** ^1^ Faculté de Médecine et d'Odontostomatologie Université des Sciences, des Techniques et des Technologies de Bamako Bamako Mali; ^2^ Service de Rhumatologie Centre Hospitalier Universitaire “Point G” Bamako Mali; ^3^ Yale University Pediatric Genomics Discovery Program New Haven Connecticut USA; ^4^ Service de Médecine Hôpital Régional “Nianankoro Fomba” Ségou Mali; ^5^ Neurogenetic Branch, NINDS, NIH Bethesda Maryland USA; ^6^ McKusick‐Nathans Institute and Department of Genetic Medicine Johns Hopkins University School of Medicine Baltimore Maryland USA; ^7^ Service de Neurologie Centre Hospitalier Universitaire “Gabriel Touré” Bamako Mali; ^8^ Service de Cardiologie Centre Hospitalier Universitaire “Gabriel Touré” Bamako Mali; ^9^ Service de Neurologie Centre Hospitalier Universitaire “Point G” Bamako Mali

**Keywords:** Africa, *COL6A1*, collagen VI‐related muscular dystrophies, Mali, splicing variant, Ullrich congenital muscular dystrophy

## Abstract

**Background:**

Congenital muscular dystrophies (CMDs) are diverse early‐onset conditions affecting skeletal muscle and connective tissue. This group includes collagen VI‐related dystrophies such as Ullrich congenital muscular dystrophy (UCMD) and Bethlem myopathy (BM), caused by mutations in the *COL6A1*, *COL6A2* and *COL6A3* genes. We report a consanguineous Malian family with three siblings affected by UCMD due to a novel homozygous splice site variant in the COL6A1 gene.

**Methods:**

After obtaining consent, three affected siblings and their relatives underwent physical examinations by specialists and laboratory tests where possible. DNA was extracted from peripheral blood for genetic testing, including Whole Exome Sequencing (WES). Putative variants were confirmed through Sanger Sequencing and assessed for pathogenicity using in silico tools.

**Results:**

The three siblings and their healthy parents, from a consanguineous marriage, presented with early‐onset progressive muscle weakness, walking difficulty, proximal motor deficits, severe muscle atrophy, hypotonia, skeletal deformities, joint hyperlaxity, ankyloses at the elbows and knees, keloid scars and dental crowding. No cardiac involvement was detected and creatine kinase (CK) levels were normal. All had low serum calcium levels, treated with oral supplements. Needle myography indicated myopathic patterns. WES identified a novel splice site variant in the first intron of COL6A1 (c.98‐1G>C), which segregated with the disease within the family. This variant is predicted to cause exon 2 skipping in *COL6A1*, with a high CADD score of 33 and Splice AI predicting it as deleterious.

**Conclusion:**

We identified a novel *COL6A1* variant in a consanguineous family, highlighting the need for further studies in larger African cohorts to enhance genetic epidemiology and prepare for future therapeutic research.

## Graphical abstract

A novel homozygous splice site variant in *COL6A1* causing Ullrich congenital muscular dystrophy in a Malian family, emphasizing the importance of genetic studies in African cohorts to advance epidemiological knowledge and therapeutic development.

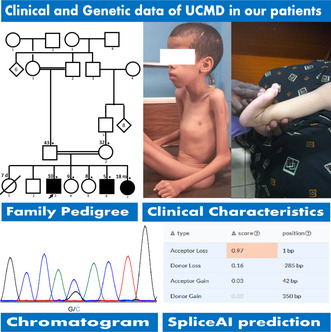

**Abbreviations**
BM: Bethlem Myopathy
CADD: combined annotation dependent depletion
CK: creatine kinase
CM: congenital myopathies
CMDs: congenital muscular dystrophies
EMG: electromyography
Fathmm: functional analysis through hidden Markov models
SS: sanger sequencing
UCMD: Ullrich congenital muscular dystrophy
WES: whole exome sequencing.

## 1 Introduction

Congenital muscular dystrophies (CMDs) are a clinically and genetically diverse group of muscular diseases that are generally characterized by early‐onset global hypotonia and muscle weakness. They are divided into subgroups, including collagen VI‐related congenital myopathies that comprise Ullrich congenital myopathy (UCMD) [MIM#254090] and Bethlem myopathy (BM) [MIM#158810]. These diseases are caused by variations in the genes coding for the major α‐chains of collagen type VI, including *COL6A1* (OMIM#120220), *COL6A2* (OMIM#120240) and *COL6A3* (OMIM#120250) (Aumailley, von der Mark, and Timpl 1985; Chu et al. 1987, 1988).

The first description of UCMD was in 1930 by Otto Ullrich, who reported it as a slowly progressive condition characterized by global hypotonia, proximal joint contractures and distal joint hyperlaxity (Ullrich 0tto. 1930). However, intelligence is usually intact in affected individuals (Bönnemann 2011).

UCMD is described as an ultrarare condition with an estimated prevalence of 0.13 per 100,000 individuals in the UK (Norwood et al. 2009). Furthermore, collagen VI‐related myopathies are now reported to be one of the most common entities under the banner of congenital muscular dystrophy (Okada et al. 2007; Peat et al. 2007). UCMD patients of African origins were described in some studies in Europe (Graziano et al. 2015), but data are scarce in Africa. In this report, we describe three affected siblings from a consanguineous marriage afflicted with UCMD and the underlying genetic defect.

## 2 Materials and Methods

This study was approved by the Ethics Committee of the Faculty of Medicine and Dentistry of Bamako, Mali. Affected siblings and their parents and relatives were enrolled after giving full consent or assent for children. Family history was recorded to draw the pedigree. A physical examination was performed by specialists (neurologists, rheumatologists, medical geneticists and cardiologists) and blood chemistries, including blood cell counts, blood ions and CK and LDH levels, were checked in all three affected siblings. Needle Electromyography was also performed in the two boys. DNA was extracted from peripheral blood from all available family members for genetic testing, including whole exome sequencing (WES). Putative variants were confirmed and checked for segregation in all available family members. Variant pathogenicity was predicted using assessment tools including Splice Vault (SV), Splice AI, ACMG score and CADD Score.

## 3 Results

**FIGURE 1:**
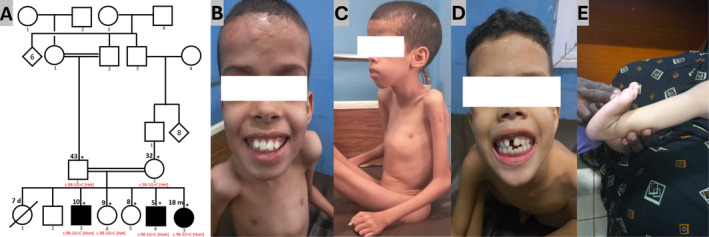
Phenotypic features of the family with *COL6A1* variant. (A) Consanguineous pedigree showing three affected probands and the healthy carrier parents and siblings. (B, C) Photographs of Proband 1 showing the keloid scars on the forehead, the Pectus carinatum and severe muscle waste in the upper body. (D, E) Photographs of Probands 2 and 3 displaying dental crowding and hyperlaxity of distal joints.

Three affected siblings (two boys and one girl) aged 10 years, 5 years and 18 months from a sibship of seven were referred to our clinic for genetic evaluation of early‐onset progressive proximal muscle weakness. They were born from asymptomatic first‐degree consanguineous parents of Arab ethnicity (Figure 1A). The first born was a girl who suddenly passed away at the age of 7 days from an unspecified disease. The inheritance pattern was suggestive of autosomal recessive. Below are the clinical findings in the three siblings.

### 3.1 Patient 1 (IV.3)

A 10‐years old male patient was seen for progressive muscle wasting. Parents report gross motor development delay. He started crawling at age 1 and was able to make only a few steps for a moment but progressively lost that ability around the age of 2 due to proximal muscle weakness and was later grounded. The symptoms worsened overtime, leading to severely reduced motor strength in four limbs, crawling became impossible. His intellectual abilities, language acquisition and social development were unremarkable. Physical examination revealed a predominantly proximal muscle weakness in both the upper and lower limbs, severe atrophy of muscles of the same area, global hypotonia and bilateral upper joint contractures and ankyloses involving the elbows and knees. Bilateral distal joint hyperlaxity was also noted in the wrists, fingers and ankles. The patient also displayed skeletal deformities, including kyphoscoliosis and pectus carinatum (Figure 1B,C). In addition, he had dental crowding and keloid scars on the forehead. Cardiovascular and pulmonary systems were uneventful. The CK levels were in the normal range (102 UI/l; range: 20–200 UI/L), but he had a low level of serum calcium (85 mg/L; range: 90–107 mg/L) and an elevated level of serum lactate (448.7 UI/l; range: 90–320 UI/L). Echocardiography was normal and needle EMG showed myogenic patterns. The patient benefited from Kinesitherapy and oral calcium supplementation.

### 3.2 Patient 2 (IV.6)

Patient 2 is the sixth child in the sibship and the third boy. He was referred to us at the age of 5 for the same reason as patient 1. Born from a normal pregnancy, he could sit at 6 months of age and walked around the age of 2. Parents noticed a wadding gait when he started walking, along with difficulties with running and jumping, leading to frequent falls. He also had difficulties climbing stairs and raising hands above his shoulders. Symptoms progressively evolved over time to being wheelchair bound. The physical examination showed that he had features like those of patient 1 but less severe. He had reduced motor strength in both upper and lower limbs with significant muscle wasting. Hyperlaxity was noted in distal joints, including wrists, fingers and ankles. In addition, he had joint contractures in the elbows and dental crowding. No facial dysmorphism or skeletal deformities were observed at this point, but he had keloid scars located on the forehead (Figure 1D). He showed no cardiovascular or respiratory symptoms. Serum CK levels were in the normal range (90 UI/L), the lactate levels were increased (606.8 UI/L) while calcium levels were low (70.7 mg/L). EMG showed myopathic features like those in his brother. His treatment consisted of physical therapy and oral calcium supplementation.

### 3.3 Patient 3 (IV.7)

An 18‐month‐old girl, last born of the sibship, was seen in our clinic for the same reasons as her older brothers. Also, born from a normal pregnancy, she started sitting at the age of 6 months and stood at the age of 12 months but could not walk unassisted. As she grew up, parents reported the same symptoms as in her brothers. Intelligence, language and social abilities are age appropriate. The physical examination was challenging but revealed more proximal than distal muscle weakness, cervical hypotonia, hyperlaxity in the wrists and ankles (Figure 1D) and bilateral pes planus. She could barely walk, even with assistance. Blood chemistry showed normal serum CK levels (93 UI/l) with high serum lactate levels (614 UI/l) and low serum calcium levels (70.5 mg/L). The EMG features were consistent with muscular dystrophy. She was put on oral calcium supplements in addition to physical therapy.

The pattern of inheritance and the clinical presentation were highly suggestive of UCMD.

**FIGURE 2:**
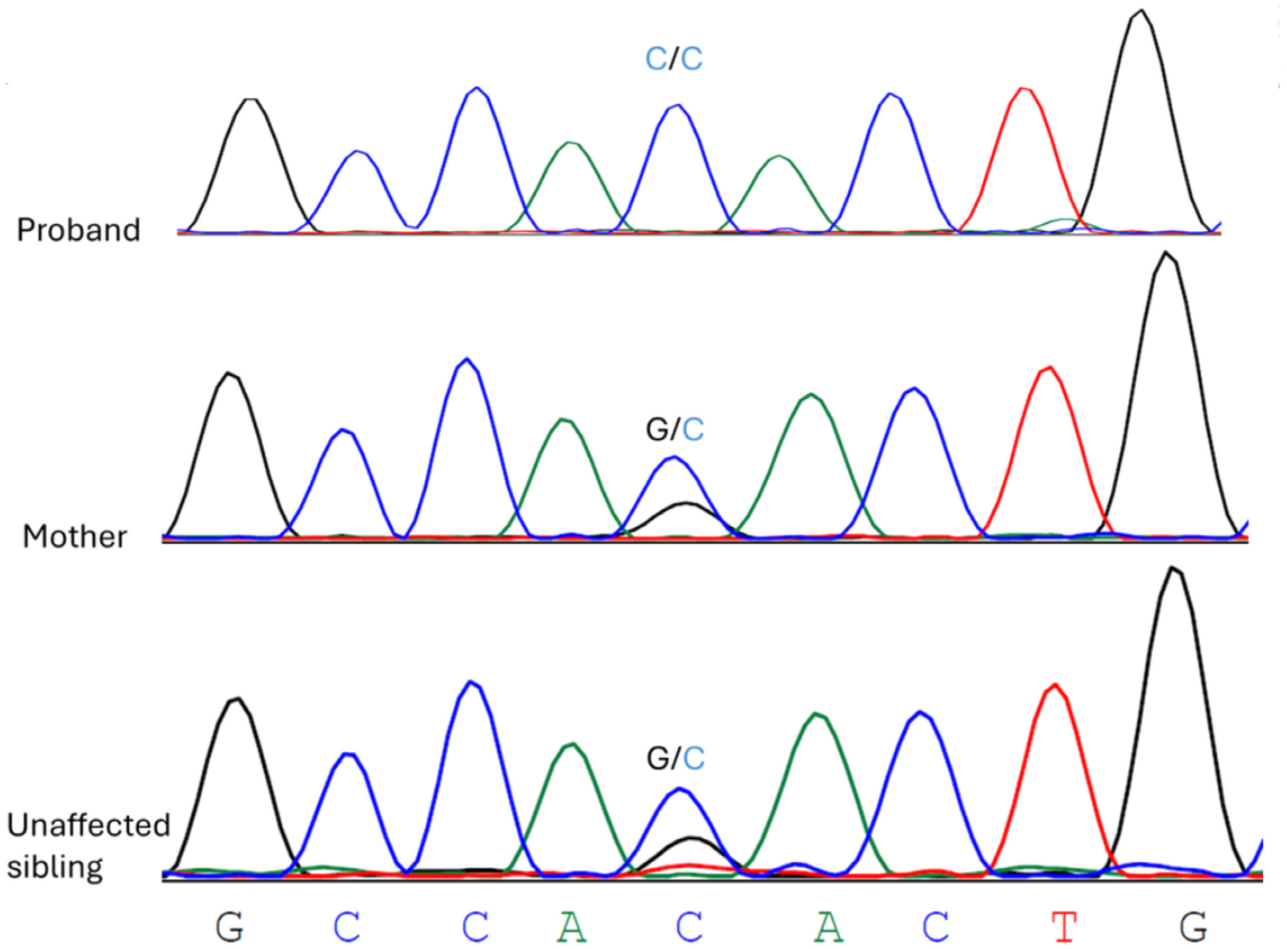
Partial chromatograms of the novel *COL6A1* c.98‐1G> C variant in the proband, the mother and one unaffected sibling.

WES identified a novel segregating, homozygous, pathogenic splicing variant (c.98‐1G> C) in intron 1 of the *COL6A1* gene (Figure 2A). This variant is predicted to be deleterious by CADD (score = 34) and to cause exon2 skipping in *COL6A1* by SpliceVault. In addition, Splice AI prediction showed a splicing acceptor loss with a high delta score = 0.97.

Details of clinical and laboratory findings are summarized in Table 1.

**TABLE 1:** A summary of the main clinical and laboratory findings.

Patients	Clinical features										Laboratory and genetic findings				Gene/variant/zygosity
	Age (years)	Gender	Age of onset (years)	Frist symptom	Hypotonia	Proximal muscle weakness	Muscle atrophy	Joints contractures	Joints hyperlaxity	Skeletal deformities	Serum CK level	Serum lactate level	Serum calcium level	EMG findings	
Proband 1	10	M	2	Walking difficulty	Present	Present	Present	Present	Present	Present	Normal	Elevated	Low	Myopathic pattern	*COL6A1*/c.98‐1G>C/Hom.
Proband 2	5	M	2	Walking difficulty	Present	Present	Present	Present	Present	Present	Normal	Elevated	Low	Myopathic pattern	*COL6A1*/c.98‐1G>C/Hom.
Proband 3	1.5	F	1	Walking difficulty	Present	Present	None	Present	Present	Present	Normal	Elevated	Low	ND	*COL6A1*/c.98‐1G>C/Hom.

Abbreviations: CK, creatine‐kinase; EMG, electromyography; F, female; Hom., homozygous; M, male; ND, not done.

## 4 Discussion and Conclusions

Here, we describe the phenotypic and genetic characteristics of three siblings from Mali with early‐onset muscular dystrophy. The clinical manifestations coupled with the family history are consistent with UCMD, the most severe form of collagen VI‐related myopathies characterized by early‐onset muscle weakness, generalized hypotonia, proximal joint contracture, distal hyperlaxity and respiratory failure (Briñas et al. 2010; Bushby, Collins, and Hicks 2014; Lamandé and Bateman 2018; Nadeau et al. 2009; Yonekawa and Nishino 2015). All probands had pronounced muscle atrophy and hypotonia, leading to delayed motor milestones and early loss of ambulation. Keloid scars were noted in patients IV.3 and IV.6, as frequently reported in UCMD patients (Nadeau et al. 2009). Patient IV.3 had kyphoscoliosis and pectus carinatum, the former being the most common type of spinal deformity reported in UCMD (Bushby, Collins, and Hicks 2014; Kim et al. 2018; Lee et al. 2017; Zanoteli et al. 2020; Zhang et al. 2022). Joint abnormalities with variable severity, including proximal joint contractures and distal joint hypermobility were also noted in the patients we describe here (Kim et al. 2018; Lee et al. 2017; Nadeau et al. 2009). Moreover, patients IV.3 and IV.6 had dental crowding, which is not typically known to be associated with UCMD. The presence of this feature does not argue against the diagnosis of UCMD, as this may be due to a totally different cause or just coincidental or stochastic. As stated in previous reports, the serum CK levels in our patients were normal (Martoni et al. 2009; Park et al. 2014). However, LDH levels were moderately high in all of them, but this is a common finding in muscular dystrophies patients (Lott and Landesman 1984; Zhu et al. 2015). Serum calcium levels were low in our patients, suggesting that the dysregulation of calcium metabolism may be involved as one of the mechanisms underlying UCMD progression (Law et al. 2020; Mareedu et al. 2021). As in previously reported cases, needle EMG showed a myopathic pattern (Bardakov et al. 2021; Elisabeth and Hh 2021; Mercuri et al. 2002).

This splicing variant has not been reported before and is classified as likely pathogenic to pathogenic according to most *in silico* prediction tools (CADD, SpliceAI) and the ACMG criteria, respectively. Interestingly, this mutation is located near a variant previously reported in Clinvar (c.98‐2_103del; rs1556423703) known to cause Bethlem Myopathy (National Center for Biotechnology Information. ClinVar; [VCV000497044.9], https://www.ncbi.nlm.nih.gov/clinvar/variation/VCV000497044.9 [accessed September 26, 2024].), suggesting that this region of the *COL6A1* gene might be a hot spot for mutations. Notably, collagens play a central role in the formation of fibrillar and microfibrillar networks within the extracellular matrix, including basement membranes, as well as in other extracellular matrix structures. The VWA domains present in these collagens facilitate protein–protein interactions (Gelse, Pöschl, and Aigner 2003). The microfibrillar type VI collagen is characterized by robust disulfide cross‐linking, contributing to a network of beaded filaments that intertwine with other collagen fibrils (Von Der Mark et al. 1984).

Although RNA samples were unavailable to verify the *COL6A1* c.98‐1G> C variant's impact on splicing, in silico tools predict that this variant likely leads to exon 2 skipping or the activation of a cryptic splice site. Exon skipping could result in an in‐frame deletion, potentially producing a shorter, dysfunctional protein (Anna and Monika 2018), while activation of a cryptic splice site might introduce a frameshift, causing premature termination and nonsense‐mediated decay (NMD) (Karousis, Nasif, and Mühlemann 2016). These outcomes would likely impair collagen VI function, consistent with the observed UCMD phenotype.

This report expands the clinical and genetic epidemiology of collagen VI‐related myopathies and opens the way to a better understanding of the phenotype–genotype correlations in UCMD.

In addition to UCMD, we considered potential differential diagnoses such as Bethlem Myopathy (BM) and Laminin α2‐related Congenital Muscular Dystrophy (MDC1A), given their overlapping clinical features of muscle weakness and contractures (Caria et al. 2019; Kwong et al. 2023; Sarkozy et al. 2020). However, genetic testing revealed no pathogenic variants in *COL6A2*, *COL6A3*, or *LAMA2*, effectively excluding these conditions. The identification of the novel *COL6A1* c.98‐1G> C variant, alongside the clinical presentation, supports this variant as the most likely cause of the siblings' phenotype. Our probands displayed unreported clinical and laboratory features, such as dental crowding and low serum calcium, suggesting that these disorders might have a unique profile in this region of Africa caused by other genetic or environmental factors or just be an expansion of the clinical presentation.

As new sequencing technologies become widely available, screening larger cohorts in these understudied populations may uncover novel findings that could shed light into the mechanism of these diseases and trigger future therapeutic research.

## Author Contributions

A.B.M., I.B., L.C., A.Y., S.T., S.D., S.D., G.L., I.A.C. designed the study, obtained the clinical information, collected the literature data and wrote the manuscript. I.P., L.C., S.H.D., C.O.G., G.L. and I.A.C. coordinated the evaluation, critically reviewed and edited the manuscript. S.D. performed the genetic testing of the family. S.D., S.B. and F.K. analyzed the sequencing data and drafted the diagnostic report. I.P., S.T., S.D. and I.A.C. were the treating rheumatologists. A.Y. performed the protein modeling, analysis and interpretation. G.L. critically revised the final manuscript for important intellectual content and approved it. All authors read and approved the final version of the manuscript.

## Ethics Statement

This study was approved by the Ethics Committee (2020/129/CE/FMOS/FAPH) of the Faculty of Medicine and Dentistry of the University of Sciences, Techniques and Technologies of Bamako. Patients and their families were enrolled in this study after giving full consent for clinical examination, genetic exploration and image taking.

## Consent

Full consent was obtained from all the participants, or from legal representatives in the case of underage patients. The signed consent forms are available upon reasonable request.

## Conflicts of Interest

The authors declare no conflicts of interest.

## Supporting information


**Figure S1.** SpliceAI prediction showing a splice acceptor loss with high delta scores (SpliceAI Δ score = 0.97).

## Data Availability

The datasets supporting the findings of this study are available from the corresponding author upon reasonable request.
