# Supplementary figures and images for: A Novel Splice Site Variant in COL6A1 Causes Ullrich Congenital Muscular Dystrophy in a Consanguineous Malian Family
Source: Mol Genet Genomic Med. 2024 Nov 11;12(11):e70032. doi: 10.1002/mgg3.70032 (PMC11551527; doi:10.1002/mgg3.70032)

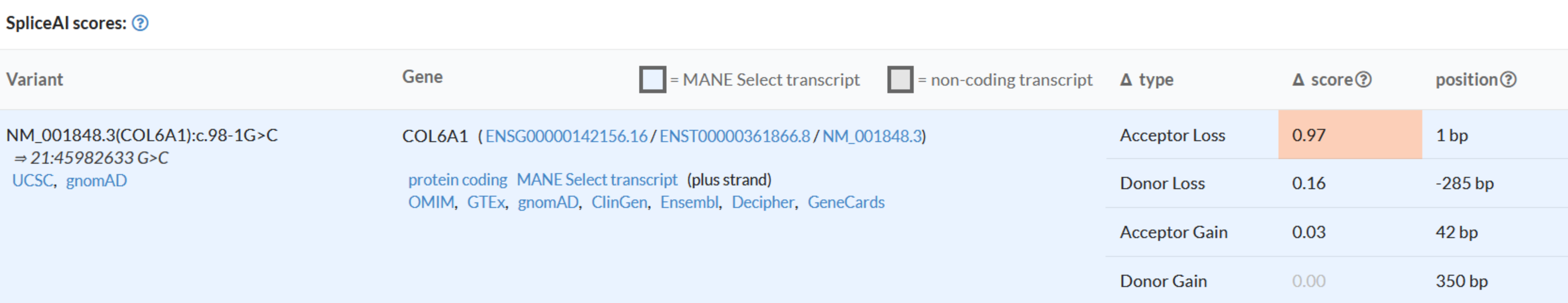

Supplement: Supplementary file 1 — Figure S1. SpliceAI prediction showing a splice acceptor loss with high delta scores (SpliceAI Δ score = 0.97). [file MGG3-12-e70032-s001.png]
